# Two novel mutations of *pfdhps* K540T and I588F, affecting sulphadoxine-pyrimethamine-resistant response in uncomplicated falciparum malaria at Banjar district, South Kalimantan Province, Indonesia

**DOI:** 10.1186/1475-2875-13-135

**Published:** 2014-04-04

**Authors:** Sukmawati Basuki, Fitriah, Sugeng Riyanto, Budiono, Yoes P Dachlan, Haruki Uemura

**Affiliations:** 1 Medical Science, Graduate School, Faculty of Medicine, Universitas Airlangga, Jl Mayjen Prof Dr Moestopo 47, Surabaya 60131, Indonesia; 2 Department of Medical Parasitology, Faculty of Medicine, Universitas Airlangga, Jl Mayjen Prof Dr Moestopo 47, Surabaya 60131, Indonesia; 3 Malaria Study Group/Laboratory of Malaria, Institute of Tropical Disease, Universitas Airlangga, Kampus C Jl Mulyorejo, Surabaya 60115, Indonesia; 4 Department of Health of Banjar District, Jl Jend A Yani KM 100, Martapura, South Kalimantan Province 70611, Indonesia; 5 Department of Public Health and Preventive Medicine, Faculty of Medicine, Universitas Airlangga, Jl Mayjen Prof Dr Moestopo 47, Surabaya 60131, Indonesia; 6 Tropical Infectious Diseases Hospital, Kampus C Universitas Airlangga, Kampus C Jl Mulyorejo, Surabaya 60115, Indonesia; 7 Department of Protozoology, Institute of Tropical Medicine, Nagasaki University, 1-12-4 Sakamoto, Nagasaki 852-8523, Japan

**Keywords:** *Plasmodium falciparum*, Sulphadoxine-pyrimethamine, *pfdhfr*, *pfdhps*

## Abstract

**Background:**

Mutations in *pfdhfr* and *pfdhps* genes have been shown to associate with sulphadoxine-pyrimethamine (SP) resistance of *Plasmodium falciparum* parasites. However, *pfdhfr*, *pfdhps* genotypes and the correlations to SP treatment outcome in Indonesia has not yet been well analysed.

**Methods:**

After obtaining informed consent, 61 uncomplicated falciparum malaria patients were recruited in Banjar district, South Kalimantan Province, Indonesia, from October 2009 to August 2010. They were treated by a single oral dose of SP and its effects on clinical and parasitological status were followed until day 28 after treatment. Occasionally, a thick smear blood film for microscopy observation and blood spot on a filter paper for *pfdhfr* and *pfdhps* genotype analysis were collected.

**Results:**

*Pfdhfr* and *pfdhps* genotypes from 24 *P. falciparum*-infected patients consisting of adequate clinical parasitological response (ACPR) (n = 6; 25.0%) and early treatment failure (ETF) (n = 10; 41.7%) or late parasitological failure (LPF) (n = 8; 33.3%) were obtained by sequencing. Two novel mutations of *pfdhps* gene, K540T and I588F, were determined in ten and five isolates, respectively. These mutations were present in the *pfdhfr*/*pfdhps* combined haplotypes of AN**RN**I/S**GTG**A (n = 6), AN**RNL**/S**GTG**A (n = 4), and AN**RN**I/S**GE**AA(588**F**) (n = 5), (mutation codons are bold typed); these haplotypes were mostly belonging to parasitological failure (ETF or LPF). The parasites acquiring five mutations in *pfdhfr*/*pfdhps* haplotypes and four mutations with additional I588F did not respond adequately to SP treatment.

**Conclusion:**

Many of *Plasmodium falciparum* infected patients in Banjar district, South Kalimantan, Indonesia did not respond adequately to SP treatment and these low ineffectiveness of SP in this area was associated with two novel mutations of *pfdhps,* K540T and I588F.

## Background

Malaria is one of the important public health problem in Indonesia, causing annual parasitic incidence of 1.85% and malarial outbreaks in several endemic areas leading 11 deaths in 2009 [1,2]. One of the major problems in Indonesia is parasite resistance to anti-malarial drugs. High rates of chloroquine resistance in falciparum and vivax malaria have been reported in Indonesia and sulphadoxine-pyrimethamine (SP) resistance in falciparum malaria was discovered in several endemic areas from 1981 to 1995. *In vitro* and *in vivo* SP resistance were found in four provinces: Central Java, East Timor, South Sulawesi, and Papua in 1991 by Tjitra *et al*. [3] and *in vitro* resistance was confirmed by using field isolates obtained from 11 malaria-endemic provinces, including East Kalimantan Province [3,4]. In spite of these reports, people in several endemic areas still commonly use SP when they are suffering from malaria because of its effectiveness, fewer side effects, low cost, and the single oral dose treatment. Additionally, SP is provided for chemoprophylaxis [5].

Efficiency of SP for falciparum malaria treatment could be predicted by analysis of *Plasmodium falciparum* target genes. Pyrimethamine and sulphadoxine inhibit the dihydrofolate reductase (PfDHFR) and dihydropteroate synthase (PfDHPS) activities of the folate biosynthesis pathway in the parasite. However, point mutations in each of the genes, *pfdhfr* and *pfdhps,* cause conformational changes in the enzyme structures and result in prevention of adequate drug binding and parasite resistance to SP. The amino acid substitutions at 16, 50, 51, 59, 108 and 164 in PfDHFR are responsible for resistance to pyrimethamine, are predicted stepwise mechanisms of mutations beginning at the codon 108 from serine to asparagine (S108N), and proceeding accumulations of N51I, C59R and I164L mutations confer high-level resistance. Similarly, resistance to sulphadoxine is induced by point mutations of the codons at amino acid positions 436, 437, 540, 581 and 613 in *pfdhps* gene, which also may progress in certain order, beginning primarily with alanine to glycine change at codon 437 (A437G), followed by K540E and A581G, then other mutations [6-14]. Mutations of *pfdhfr* and *pfdhps* have also correlated with SP resistance *in vivo* by triple mutant of *pfdhfr* at positions N51I, C59R, S108N and of quintuple mutant of *pfdhfr/pfdhps* combination with additional mutant of *pfdhps* at positions A437G and K540E [9,15-20].

In Indonesia, there are not many reports about genotype analysis of *pfdhfr* and *pfdhps* and their correlation with SP treatment outcome. Nagesha *et al*. reported in 2001 with West Papua parasites obtained in 1996–99 that polymorphisms at C59R and S108N of *pfdhfr,* and at A437G and K540E of *pfdhps* were closely associated with resistance [21]. They mentioned no mutations were observed at codons 16, 50, 51 and 164 of the *pfdhfr* gene and at 436, 581 and 613 of the *pfshps* gene [21]. Syafruddin *et al.* expanded the analysis to eight malaria-endemic areas, representing a broad region of the eastern and western Indonesian archipelago and presented additional polymorphisms in *pfdhfr* gene at the codons of A16V and S108T from several island parasite samples, the more common distributions being among eastern parts [22]. In this report, they mentioned less frequency of *pfdhps* polymorphisms; most of the parasites presented wild type *pfdhps* and about 15% of 437G; less than 5% of K540E mutations were detected [22]. Recently analysed *pfdhfr* and *pfdhps* genotypes, using a small number of parasite samples from South Kalimantan, by PCR-RFLP method were presented with complete mutations at S108N of *pfdhfr* and A437G of *pfdhps*, in addition *pfdhps* K540E substitution from one patient’s parasites [23].

In this study, sequence polymorphisms of *pfdhfr* and *pfdhps* genes, and these correlations to SP treatment outcome were conducted in 2009–10 in South Kalimantann Province, Indonesia. It will be helpful to estimate SP effectiveness in many situations.

## Methods

### Study site and participants

**Figure 1:**
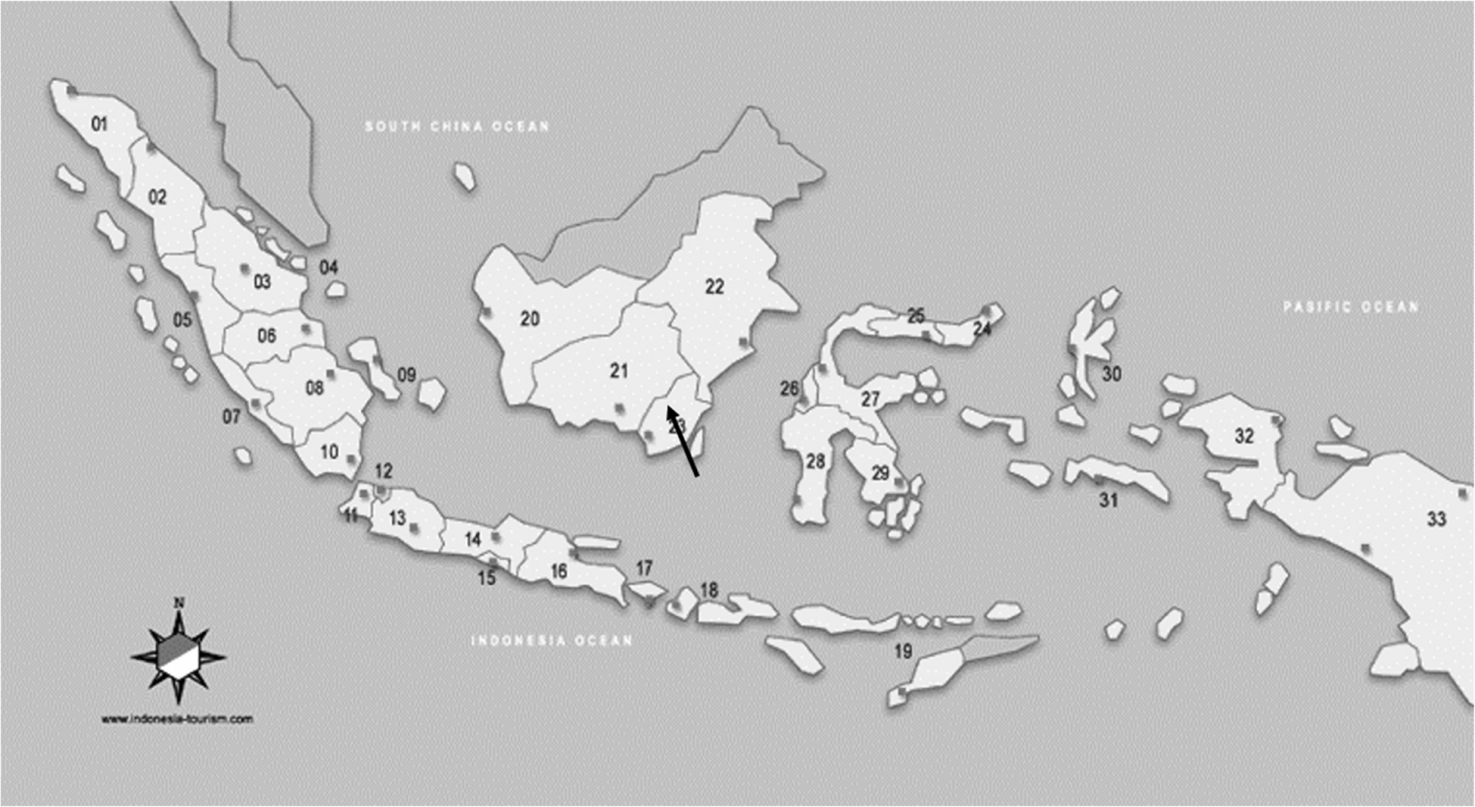
Study site, Banjar district, South Kalimantan Province, Indonesia.

The study was conducted at Sei Pinang and Aranio subdistricts, Banjar district, South Kalimantan Province, Indonesia (Figure 1) from October 2009 to August 2010, and was approved by the Ethical Committees, Faculty of Medicine, Universitas Airlangga, Surabaya, Indonesia and Institute of Tropical Medicine, Nagasaki University, Japan. Total population of the study site subdistricts was 25,975 in 2008 and the most common occupations were farmers and mining workers. Both subdistricts have high prevalence of malaria. Written informed consent was obtained from each participant, or from caretakers if participants were under 12 years of age, after explanation in the local language of the study procedure.

Symptomatic falciparum malaria patients without complications were recruited by local study teams, which consisted of nurses and microscopists from the regional health centre. Both *P. falciparum* alone and *P. falciparum* infections mixed with other malaria parasites, diagnosed by Now® Malaria ICT diagnosis kit (Binax Inc, Portland, ME, USA), were selected from uncomplicated falciparum malaria patients and treated with a single oral dose of 25 mg/kg sulphadoxine: 1.25 mg/kg pyrimethamine tablet (PT Kimia Farma Tbk. DOEN (Daftar Obat Esensial Nasional)), which was contributed by Ministry of Health, Indonesia. Patients took the medicine in front of a member of the team. Patient follow-up was conducted by the teams, by either a team member visiting a patient at home or the patient visiting the health centre, for the purpose of obtaining information about clinical improvement or side effects of the treatment, for 28 days, as described by WHO [24]. On each occasion, a finger-prick blood sample was taken to prepare a thick blood smear film for microscopical observation and a filter-paper blood spot for DNA extraction (on day 7 and day 21). Patients that failed treatment with SP were treated with a combination of quinine and primaquine on day 28. Treatment outcomes were assessed using the 2009 WHO standard definition for clinical and parasitological responses: adequate clinical parasitological response (ACPR), early treatment failure (ETF), late clinical failure (LCF), late parasitological failure (LPF) [25].

### *Plasmodium falciparum* isolates

#### Microscopy

Thick smear blood films stained with Giemsa solution (Merck, Germany) were performed for *P. falciparum* diagnosis. The parasite density was determined by counting asexual parasites in each 200 white blood cells, then converted as the number of asexual parasites per μl blood with consideration of an assumed white blood cell density (8 000 per μl) [26].

#### Mutation analysis

Parasite DNA was isolated from the dried blood spots on filter paper (Advantec, Toyo Roshi Kaisha Ltd, Japan) using QIAamp DNA blood mini kit (Qiagen, the Netherlands) as described by Sakihama *et al.*[27]. *Plasmodium falciparum* was identified by nested PCR technique using primers of Snounou *et al*. and Kimura *et al.*[28,29]. *Pfdhfr* and *pfdhps* genotypes were determined by sequence analysis as previously described by Isozumi *et al.*[30]. The amplified PCR products were directly used as templates for sequencing with a BigDye Terminator (version 1.1) cycle sequencing kit and a model 3730 genetic analyzer (Applied Biosystems, USA). Alleles at residues 16, 51, 59, 108, and 164 of the *pfdhfr,* and at residues 436, 437, 540, 581, and 613 of the *pfdhps* were read carefully, and two independent PCR products were subjected to sequence analysis in the case of new or rare mutations.

#### Statistical analysis

Data were entered in Microsoft Excel and exported to SPSS version 17.0 for analysis. The strength of an association was evaluated by calculating odds ratios (OR). Chi-square and Fisher’s exact tests were used, where applicable, to assess the relationship between single and multiple mutations to treatment outcomes. Arithmetical means, median and percentage were calculated, where applicable, for subject characteristics and treatment outcomes. Allele proportions were calculated as the number carrying a certain allele divided by the number of samples with positive PCR outcomes.

## Results

### Patient characteristics and treatment outcomes

A total of 87 malaria patients were identified at primary health centres. However, 22 malarial patients were unwillingness to participate in this study, one participant dropped out from follow-up on day 2 after treatment because of moving from the study site, and three vivax malaria patients were excluded. Thus, a total of 61 falciparum malaria patients treated with SP, who had characteristics as presented in Table 1, were included in this analysis. One malaria patient with mixed infections, which were identified by microscopy and PCR as falciparum and vivax malaria, was involved in the study.

**Table 1:** Subject characteristics and treatment outcomes

**Characteristics**	**No of patients (n = 61)**	
Median age in years (range)	25 (14–55)	
Male: Female	49: 12	
Mean parasite density of D0 (per μl) (range)	2,835 (40–38,368)	
Adequate clinical parasitogical response (%)	22 (36.1)	
Parasitological failure* (%)	39 (63.9)	
Early treatment failure (ETF)	16 (26.2)	
Late parasitological failure (LPF)	23 (37.7)	
Late clinical failure (%)	0	
Parasite clearance** (%):	A)	B)
On D3	18 (29.5)	15 (24.6)
On D7	22 (36.1)	20 (32.8)
On D14	35 (57.4)	32 (52.5)
On D21	40 (65.6)	38 (62.3)
On D28	47 (77.0)	43 (70.5)

*Parasitological failure is either early treatment failure or late parasitological failure cases. The actually detected parasites were few in number in many cases and it makes some cases difficult to determine whether ETF or LPF clearly.

**Parasite clearance was determined by microscopy, when A) the cases that an asexual parasite was not detected even gametocytes were observed on the day after treatment; B) the cases that neither asexual nor sexual parasites were detected on the day after treatment. D0 = on the day of treatment.

SP treatment resulted in 36.1% of ACPR and 63.9% of parasitological failures; 26.2% of ETF and 37.7% of LPF. Late clinical failure were not observed in this study (Table 1) and no adverse reactions were identified. Although there were many cases of ETF and LPF, the actually detected parasites were few in number in many cases and the parasite-positive rates were decreasing gradually from 39 microscopically positive cases (63.9%) on day 7 to 14 cases (23.0%) on day 28 (Table 1, presented as % of clearance cases). In these cases, the sexual stage parasites were also disappearing consistently and 43 cases (70.5%) were negative in both asexual and sexual stage parasites on day 28.

### *Pfdhfr* and *pfdhps* genotypes

The *pfdhfr* and *pfdhps* genotypes were obtained by DNA sequencing of PCR products from *P. falciparum*-infected patients on the day before treatment (D0) and those from parasite positives on the day after treatment (D7 and D21). The whole set genotypes of *pfdhfr* and *pfdhps* were assessed from 27 patients of *P. falciparum* infection on D0, from 15 blood samples of D7 and 18 samples of D21. Based on microscopical observation and genotype analysis, correlations between the parasite genotypes and responses to SP treatment were presented on Table 2. The cases of mixed genotype infections (cases A and C), or different genotypes of *pfdhfr* or *pfdhps* on day 0 and the following days (cases B and C), are presented separately (Table 3). In total, *pfdhfr* and *pfdhps* genotypes of 31 *P. falciparum* parasites were obtained for analysis.

**Table 2:** **Correlation between *pfdhfr*/*pfdhps *** **genotypes and treatment outcomes to sulphadoxine-pyrimethamine**

***pfdhfr*** */pfdhps** **genotypes**	**Prevalence (%) (n = 24)**	**Treatment outcomes**		
		**ACPR**	**ETF**	**LPF**
ANC**N**I/SAKAA	1 (4.1)	1		
AN**RN**I/S**GE**AA	4 (16.7)	2	1	1
AN**RN**I/S**GE**AA(588**F**)	5 (20.8)	1	2	2
AN**RN**I/S**GTG**A	6 (25.0)		2	4
AN**RNL**/S**G**KAA	4 (16.7)	2	1	1
AN**RNL**/S**GTG**A	4 (16.7)		4	

*pfdhfr genotypes are presented at amino acid positions of 16, 51, 59, 108, and 164.

**pfdhps genotypes are presented at amino acid positions of 436, 437, 540, 581, and 613 (and 588).

The bold type indicates amino acid substitution.

ACPR = adequate clinical parasitological response; LPF = late parasitological failure, ETF = early treatment failure.

**Table 3:** **Cases with different genotypes of *pfdhfr *** **and *pfdhps *** **on the day before drug regimen (D0) and on the days after treatment (D7 and D21)**

	***pfdhfr*** */pfdhps** **genotypes**		
**Case**	**D0**	**D7**	**D21**
A	ANC**N**I/S**G**K**G**A	-	-
	AN**RN**I/S**GE**AA(588**F**)		
B	ANC**N**I/S**G**K**G**A	AN**RN**I/S**GTG**A	AN**RN**I/S**GTG**A
C	AN**RNL**/S**G**KAA	AN**RN**I/S**GE**AA(588**F**)	AN**RN**I/S**GE**AA(588**F**)
		AN**RN**I/S**G**K**G**A	AN**RN**I/S**G**K**G**A

*pfdhfr genotypes are presented at amino acid positions of 16, 51, 59, 108, and 164.

**pfdhps genotypes are presented at amino acid positions of 436, 437, 540, 581, and 613 (and 588).

The bold type indicates amino acid substitution.

**Figure 2:**
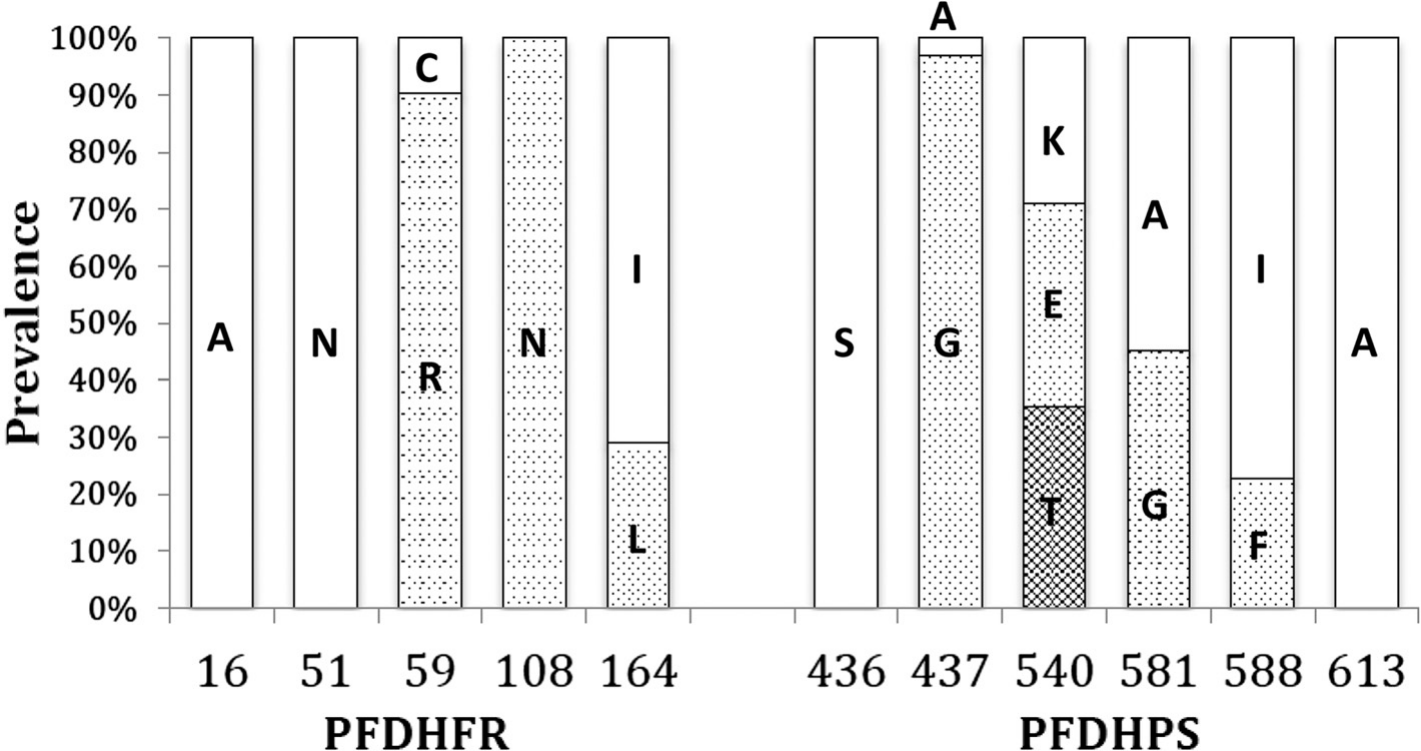
**Prevalence of the mutation *pfdhfr *and *pfdhps *genotypes.** The horizontal axis shows the position or codon of the mutation *pfdhfr* and *pfdhps* genes. The white box indicates the wild type and the texture boxes are mutants written by amino acid abbreviations. A = alanine, N = asparagine, C = cysteine, R = arginine, I = isoleucine, L = leucine, S = serine, G = glycine, K = lysine, E = glutamic acid, T = threonine, F = phenylalanine.

The *pfdhfr* mutations at amino acid positions of Cys59, Ser108 and Ile164 to Arg (90.3%), Asn (100%) and Leu (29%), respectively were detected (Figure 2). All the parasites carried the S108N substitution and no wild type *pfdhfr* allele ANCSI (at positions 16, 51, 59, 108, and 164, respectively) was obtained in this study. In total, three different haplotype alleles, ANC**N**I, AN**RN**I and AN**RNL** (bold type indicates the mutated alleles) were detected in this area (Tables 2 and 3). This simple accumulation pattern of *pfdhfr* mutation genotypes supports the stepwise mechanism of resistant gene evolution hypothesis.

In the *pfdhps* gene, mutations were detected at the positions of Ala437, Lys540, Ala581 and Ile588 to Gly (96.8%), Glu or Thr (35.5, 35.5%), Gly (45.2%), and Phe (22.6%), respectively. No other substitutions, including at Ser436 and Ala613, were observed (Figure 2). The high proportion of mutation was detected at the position of Ala437 to Gly, which is regarded as the first and the most important change required for sulphadoxine resistance. Two types of mutation were determined at amino acid position 540; AAA (Lys codon) was changed to GAA (Glu) or ACA (Thr) by different types of single nucleotide substitutions. Lysine (K) to glutamic acid (E) substitution at position 540 is reported from many malaria-endemic areas, however, threonine (T) substitution is unique to *P. falciparum* parasites in South Kalimantan. In addition, the other new substitution was identified at amino acid position 588, which ATT (Ile codon) converted to TTT (Phe codon) by a single point mutation is not previously reported. Altogether including these two novel mutations, six *pfdhps* haplotypes, SAKAA (at positions 436, 437, 540, 581, and 613, respectively), S**G**KAA, S**GE**AA, S**G**K**G**A, S**GTG**A and S**GE**AA(588**F**) were determined as a single haplotype infection or multiple haplotype infection cases (Tables 2 and 3). The wild type *pfdhps* allele was present in one case at this study area. The genotypes containing two new mutations were present as the precise haplotypes separately; K540T exists as S**GTG**A only and I588F mutation was detected in specific haplotype S**GE**AA(588**F**). It suggests these new mutations were introduced independently in recent times.

### *Pfdhfr*, *pfdhps* genotypes and SP treatment outcomes

With a combination of three *pfdhfr* and six *pfdhps* alleles, eight different combined *pfdhfr*/*pfdhps* haplotypes were determined. Six combined haplotypes from a single genotype infection patient are presented in Table 2. Two additional combined *pfdhfr*/*pfdhps* haplotypes, ANC**N**I/S**G**K**G**A and AN**RN**I/S**G**K**G**A, were detected in multiple genotype infection cases (Table 3). Each case of SP treatment outcome is summarized and presented with a base of combined haplotypes (Table 2).

Although the actually detected parasites were few in number in many cases and it makes such cases difficult to determine whether ETF or LPF clearly, some correlations between *pfdhfr*/*pfdhps* haplotypes and SP sensitivity were observed. The parasites containing ANC**N**I/SAKAA and ANC**N**I/S**G**K**G**A combination haplotypes on day 0 were not detectable on day 7 after treatment and these belonged to ACPR. In the quadruple mutant case of AN**RN**I/S**GE**AA, AN**RN**I/S**GE**AA(588**F**) and AN**RNL**/S**G**KAA haplotypes (not included new I588F mutation in counting number of mutations), both ACPR and parasitological failure responses (ETF or LPF) were observed. All cases of parasites presenting AN**RN**I/S**GTG**A and AN**RNL**/S**GTG**A haplotypes, both of which contain new K540T substitution, on day 0 were not cleared on day 7 and these were included in a group of parasitological failure (ETF or LPF); the former quintuple mutant was ETF or LTF and the latter sextuple case was all ETF. The quintuple or more mutant parasites acquiring more than five mutations in *pfdhfr*/*pfdhps* haplotypes did not respond adequately to SP treatment (Table 4). The new I588F mutation was observed only in the parasites of AN**RN**I/S**GE**AA(588**F**) haplotype and comparison of those with or without I588F mutation indicates this amino acid substitution contributes insufficient response of the parasites to SP treatment (Tables 2 and 3).

**Table 4:** **Assessment association between *pfdhfr *** **and *pfdhps *** **genotypes and treatment outcomes**

**Genotype**	**Treatment outcomes**		**95% CI**	***P***
	**ACPR**	**ETF/LPF**		
< quintuple mutants	6	8	1.112-2.755	0.024
≥ quintuple mutants	0	10		
Total	6	18		

Quintuple mutants = the cases containing five mutations of *pfdhfr* 108N, 59R, 164L and *pfdhps* 437G, 540E or 540T, 581G.

ACPR = adequate clinical parasitological response; ETF/LPF = parasitological failure.

In this follow-up study of SP treatment, different parasite genotypes were determined at day 0 and day 7, 21 in two cases (Table 3, B and C). The parasite numbers decreased at day 3 were increased at day 7 in these two cases (data not shown); it suggested a possibility that the increases in parasite numbers were due to new infections and lower responses of these parasites to SP. Indeed both B and C cases, the quintuple mutant AN**RN**I/S**GTG**A in B and the quadruple mutant AN**RN**I/S**GE**AA(588**F**) in C belong the genotypes that were not respond adequately to SP treatment in Table 2.

## Discussion

In this report, polymorphisms of *pfdhfr* and *pfdhps* genes and these correlations with SP treatment outcomes in South Kalimantan, Indonesia were analyzed. The codons at amino acid positions of 16, 51, 59, 108 and 164 in *pfdhfr* gene, and 436, 437, 540, 581 and 613 in *pfdhps* gene were investigated by DNA sequencing of PCR products from *P. falciparum*-infected patient blood. Amino acid substitutions of these polymorphic positions have been shown to be important for SP resistance [6,8,20,31,32] and mutations at several positions, C59R, S108N and I164L in *pfdhfr*, A437G, K540E or T, A581G, and additionally I588F in *pfdhps* gene were observed in this research. In these mutations, K540T and I588F of *pfdhps* are unique in this study area. With these polymorphisms in *pfdhfr* and *pfdhps* genes, three different *pfdhfr* and six *pfdhps* haplotype alleles were determined, and then a total of eight *pfdhfr*/*pfdhps* combined haplotypes were assessed. The comparison of the haplotypes supported the stepwise accumulation of mutations in *pfdhfr* and *pfdhps* alleles and that it might be caused by intensive use of SP for malaria treatment at the study sites.

The mutation of K to E at position 540 of *pfdhps* had strong correlation with resistance to sulphadoxine and to SP treatment [6,10,11,33-35]. The novel mutation K540T of *pfdhps* at the same amino acid position was identified in this study. It was shown that *P. falciparum* parasites carrying the K540T mutation of *pfdhps* allele were all found in Parasitological failure, ETF or LPF (Table 2), and each isolate carrying K540T was always associated to the mutations of A437G and A581G, forming the triple mutant of *pfdhps* allele. This accumulation of mutations causes parasite resistance to SP treatment. It also shows the second novel mutation at the position *pfdhps* 588, I to F (I588**F**). This mutation was always observed in association with mutations A437G and K540E forming double mutant of *pfdhps* (the I588F mutation is not included in counting number of mutations). Most of the parasites presenting the I588F mutation caused Parasitological failure. These findings indicate that these novel mutations could be a predictor of resistance to SP treatment. The different amino acid substitutions at the same position may affect different levels in sulphadoxine inhibition, such that K540N *pfdhps* brings a lower level of sulpha drug resistance than the mutation K540E [34]. An analysis study of how strong the K540T mutation in *pfdhps* affects the parasite response to sulphadoxine is required.

The K540T mutation is recently reported in Sabah, Malaysian Borneo, by Lau *et al*. [36]. The same unique mutant K540T and S**GTG**A genotype of *pfdhps* were found in this study site in South Kalimantan, Indonesia and in Sabah, Malaysian Borneo; these two regions are located on the same island which suggests that the K540T mutation originates on the island or was introduced, as the parasites expressing it, S**GTG**A genotype of *pfdhps,* then spread on the island. In the case of Malaysian Borneo, the parasites carrying K540E, previously predominant point mutation at position 540, was substituted by K540T and the S**GTG**A type allele of *pfdhps* gene became the most prevalent allele in that region. In the case of South Kalimantan, it is noteworthy that I588F, the other new mutation in *pfdhps* and which may contribute to parasite resistance to SP, exists additionally. The origin and spread of these two novel *pfdhps* mutations in Indonesia is now under investigation.

In this report, eight *pfdhfr*/*pfdhps* combined haplotypes were observed in South Kalimantan, Indonesia and these correlations to SP treatment outcome were presented; more than 63% parasites did not respond to SP adequately and correlations between *pfdhfr*/*pfdhps* genotypes and parasite response to SP were summarized. The parasites presenting less than three mutations in *pfdhfr*/*pfdhps* combined haplotypes responded adequately, and in the cases of four mutations in combined haplotypes (I588F mutation is not included for counting the number of mutations), the parasite responses were not consistent, either ACPR, ETF or LPF (Table 2). The parasites harbouring more than five mutations in *pfdhfr*/*pfdhps* combined haplotypes did not always respond sufficiently as reported previously [20]. Even though a limitation in this study that the drug levels in the blood were not measured, the correlations *pfdhfr*/*pfdhps* haplotypes and SP treatment outcome could be applicable in other areas to predict SP efficiency in Indonesia.

According to Nagesha and colleagues [21,22], Fansidar (Roche Pharmaceuticals SP)-resistant parasites from West Papua and some areas in Indonesia presented *pfdhfr/pfdhps* genotypes of 59R + 108N/437G + 540E. This quadruple *pfdhfr*/*pfdhps* combined haplotype observed in this study was also responding either ACPR, ETF or LPF to SP, and it can be understood that the quadruple *pfdhfr*/*pfdhps* haplotype is at the beginning of SP resistance. Nagesha *et al*. did not detect resistant parasites expressing quintuple combined haplotypes at the time of parasite collection, but it is important to analyse the current situation in West Papua and other islands.

It has been investigated whether either *pfdhfr* or *pfdhps* genotype has a principal role in parasite resistance to SP treatment [11,33,35,37]. The present study and previous works have demonstrated the genotype of *pfdhfr* is not complicated and the *pfdhps* is more polymorphic in Indonesian parasites. In the cases of the Kalimantan sample analysis, most of the parasites (90.3%) harbouring more than two mutations in *pfdhfr* gene were associated with different types of *pfdhps* alleles, S**GE**AA, S**GE**AA(588**F**) and S**GTG**A, and which are critically important for parasite resistance to SP treatment. The parasites of K540E could be aware of a situation at the beginning of SP resistance, and with an additional I588F mutation tend to some resistance (Table 2). The other K540T substitution is associated with additional mutation A581G and is recognized as SP-resistant (Table 2). Altogether, the genotype of *pfdhps*, particularly K540E or T and I588F are important for understanding the parasite SP responses.

The polymorphisms and genotyping of *P. falciparum* drug-resistant genes are in many cases determined by using specific restriction enzyme digestion. In the case of *pfdhps* I588F mutation, ATT (Ile codon) to AAA (Phe codon) could be distinguished by Dra I digestion; the mutation AAA (Phe) is digested. Unfortunately, it was not possible to identify *pfdhps* K540T by a simple restriction enzyme digestion. The detection systems to identify single nucleotide differences, such as using hybridization of fluorescence-labelled oligonucleotide, comparison of melting temperature curves of PCR products, or sequencing is required. However, all the *pfdhps* K540T mutation were detected in the S**GTG**A genotype of *pfdhps* and A581G mutation by PCR-RFLP methodology [38] could be applied as an indirect indication signal for the mutation.

SP should not be considered for therapeutic use in this region of Indonesia. SP efficiency in this study area was only 36.1% and remaining 63.9% presented parasites more than one week after treatment. In either ETF or LPF cases, patients did not present severe symptoms and the parasites in the blood were decreasing gradually. It might be one of the reasons local people kept and used SP for malaria treatment.

## Conclusion

Polymorphisms of *pfdhfr*/*pfdhps* genotypes in Banjar district, South Kalimantan, Indonesia, and these correlations with parasite responses to SP treatment were analyzed in this study. The *pfdhfr*/*pfdhps* combined haplotypes were clearly related to the treatment outcomes and the two newly found novel *pfdhps* mutations, K540T and I588F, were important indicators of SP resistance.

## Competing interests

The authors have declared that they have no competing interests.

## Authors’ contributions

SB and HU prepared the protocol, analysed and interpreted data, wrote the paper; SR coordinated field study and data collection in location; F analysed samples at the laboratory; B developed protocol; YPD reviewed the protocol, results, interpretation and the manuscript. All authors read and approved the final manuscript.
